# Histologic Activity in Inflammatory Bowel Disease and Risk of Serious Infections: A Nationwide Study

**DOI:** 10.1016/j.cgh.2023.10.013

**Published:** 2023-10-30

**Authors:** Karl Mårild, Jonas Söderling, Jordan Axelrad, Jonas Halfvarson, Anders Forss, SWIBREG Study GroupOla Olén, Jonas F. Ludvigsson

**Affiliations:** 1 Department of Pediatrics, Institute of Clinical Sciences, Sahlgrenska Academy, Gothenburg, Sweden; 2 Department of Pediatrics, Queen Silvia Children’s Hospital, Gothenburg, Sweden; 3 Department of Medical Epidemiology and Biostatistics, Karolinska Institutet, Solna, Sweden; 4 Clinical Epidemiology Division, Department of Medicine Solna, Karolinska Institutet, Stockholm, Sweden; 5 Inflammatory Bowel Disease Center at New York University Langone Health, Division of Gastroenterology, Department of Medicine, New York University Grossman School of Medicine, New York, New York; 6 Department of Gastroenterology, Faculty of Medicine and Health, Örebro University, Örebro, Sweden; 7 Gastroenterology Unit, Department of Gastroenterology, Dermatovenereology and Rheumatology, Karolinska University Hospital, Stockholm, Sweden; 8 Pediatric Gastroenterology Unit, Sach’s Children and Youth Hospital, Stockholm South General Hospital, Stockholm, Sweden; 9 Department of Clinical Science and Education Södersjukhuset, Karolinska Institutet, Stockholm, Sweden; 10 Department of Pediatrics, Örebro University Hospital, Örebro, Sweden; 11 Department of Medicine, Columbia University College of Physicians and Surgeons, New York, New York

**Keywords:** Histology, Population-Based, Infections

## Abstract

**BACKGROUND & AIMS:**

Individuals with inflammatory bowel disease (IBD) are at increased risk of serious infections, but whether this risk varies by histologic disease activity is unclear.

**METHODS:**

This was a national population-based study of 55,626 individuals diagnosed with IBD in 1990 to 2016 with longitudinal data on ileocolorectal biopsy specimens followed up through 2016. Serious infections were defined as having an inpatient infectious disease diagnosis in the Swedish National Patient Register. We used Cox regression to estimate hazard ratios (HRs) for serious infections in the 12 months after documentation of histologic inflammation (vs histologic remission), adjusting for social and demographic factors, chronic comorbidities, prior IBD-related surgery, and hospitalization. We also adjusted for IBD-related medications in sensitivity analyses.

**RESULTS:**

With histologic inflammation vs remission, there was 4.62 (95% CI, 4.46–4.78) and 2.53 (95% CI, 2.36–2.70) serious infections per 100 person-years of follow-up, respectively (adjusted HR [aHR], 1.59; 95% CI, 1.48–1.72). Histologic inflammation (vs remission) was associated with an increased risk of serious infections in ulcerative colitis (aHR, 1.68; 95% CI, 1.51–1.87) and Crohn’s disease (aHR, 1.59; 95% CI, 1.40–1.80). The aHRs of sepsis and opportunistic infections were 1.66 (95% CI, 1.28–2.15) and 1.71 (95% CI, 1.22–2.41), respectively. Overall, results were consistent across age groups, sex, and education level, and remained largely unchanged after adjustment for IBD-related medications (aHR, 1.47; 95% CI, 1.34–1.61).

**CONCLUSIONS:**

Histologic inflammation of IBD was an independent risk factor of serious infections, including sepsis, suggesting that achieving histologic remission may reduce infections in IBD.

## Graphical Abstract

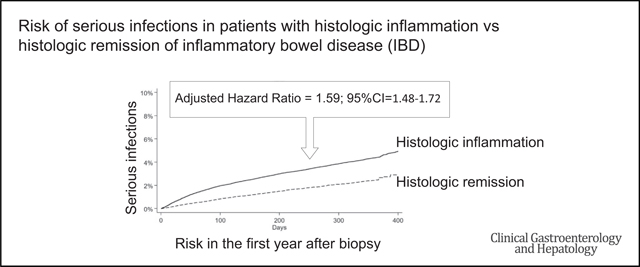

Inflammatory bowel disease (IBD), consisting mainly of Crohn’s disease (CD) and ulcerative colitis (UC), is a lifelong condition characterized by episodes of remission and relapse. Beyond clinical and endoscopic remission, histologic remission (ie, the absence of structural changes, inflammation, and ulceration/erosion) has emerged as an aspirational therapeutic goal in IBD, particularly in UC.^1–3^

Individuals with IBD are at an increased risk of serious infections,^4,5^ which represent an important cause of death in children and adults with the disease.^6,7^ On the one hand, surgical and medical therapy for IBD may predispose to infections.^8^ On the other hand, there is a notion that disease activity alone increases susceptibility to infections, but with limited data to support such claims. Most studies also have lacked refined exposure assessments to disentangle disease activity from medical therapy.^9^ Despite the increasing appreciation of histological activity in IBD, its association with serious infections is unknown. Current IBD guidelines do not address histologic inflammation as a risk factor for serious infections.^8^

Taking advantage of nationwide IBD cohort data with longitudinal biopsy data,^10^ we aimed to determine the association between histologic IBD activity and the risk of serious infections overall and across infection categories after accounting for medical therapy and disease severity.

## Methods

### Study Sample

This study was based on the nationwide Epidemiology Strengthened by histoPathology Reports in Sweden (ESPRESSO) cohort,^10^ for which all computerized gastrointestinal histology reports from Sweden’s 28 pathology departments have been linked to National Health Care Registers. We defined IBD as having either 2 or more International Classification of Diseases (ICD) codes in the Swedish National Patient Register (NPR)^11^ or 1 or more ICD codes and 1 or more relevant ileocolorectal histopathology (Systematized Nomenclature of Medicine [SNOMED]) codes (Supplementary Table 1). The NPR, started in 1964, gained nationwide inpatient coverage in 1987, and since 2001 also contains hospital-based outpatient care.^11^ On medical record review, our IBD definition has shown a positive predictive value of 93% to 95% for a clinical diagnosis of IBD.^12,13^ We used subtype-specific ICD codes to classify UC, CD, and IBD-unclassified, a subtype in which UC cannot be readily distinguished from CD and vice versa. In line with previous works,^14–16^ IBD patients were characterized according to IBD-related surgery and the extent and location^17^ of the disease at diagnosis (Supplementary Tables 2 and 3).

This study comprised 55,626 unique individuals diagnosed with IBD in 1990 to 2016 and an ileocolorectal biopsy. As described in Supplementary Table 4, we used linked data from the NPR and the National Cancer Register to exclude individuals who, within 5 years from the index date (ie, the start of the exposure period), had been diagnosed with any cancer, chronic infectious disease (eg, tuberculosis or hepatitis B), or had undergone organ transplantation (Supplementary Table 5).

Our main analyses also excluded individuals with any diagnosis of inpatient infectious disease within 5 years from the index date to reduce the risk that unevenly distributed susceptibility to serious infections may affect our estimates (see Supplementary Table 6 for ICD codes). Sensitivity analyses were performed without applying this exclusion criterion.

### Exposure: Histologic inflammation Vs Histologic Remission

Motivated by consensus reports,^2,3^ histologic inflammation was defined as 1 or more histopathology (SNOMED) codes for ileocolorectal inflammation (acute or chronic) or ulceration/erosion, as detailed in Supplementary Table 7. This definition of histologic appearance has been used previously to examine the risk of adverse pregnancy outcomes in female patients with IBD.^18^ Histologic remission was defined through the presence of ileocolorectal histopathology (SNOMED) codes for normal mucosa (M00100/M00110),^10^ the absence of SNOMED codes for inflammation (acute or chronic), and ulceration/erosion (Supplementary Table 7). Histologic inflammation vs remission was defined based on the worst histologic appearance across all ileocolorectal segments and did not rely on laboratory markers or endoscopic appearance. Histologic scoring systems of IBD^19^ are not used routinely in Sweden. Stratified analyses were performed based on the extent and location of the disease at diagnosis.

**Figure 1.**
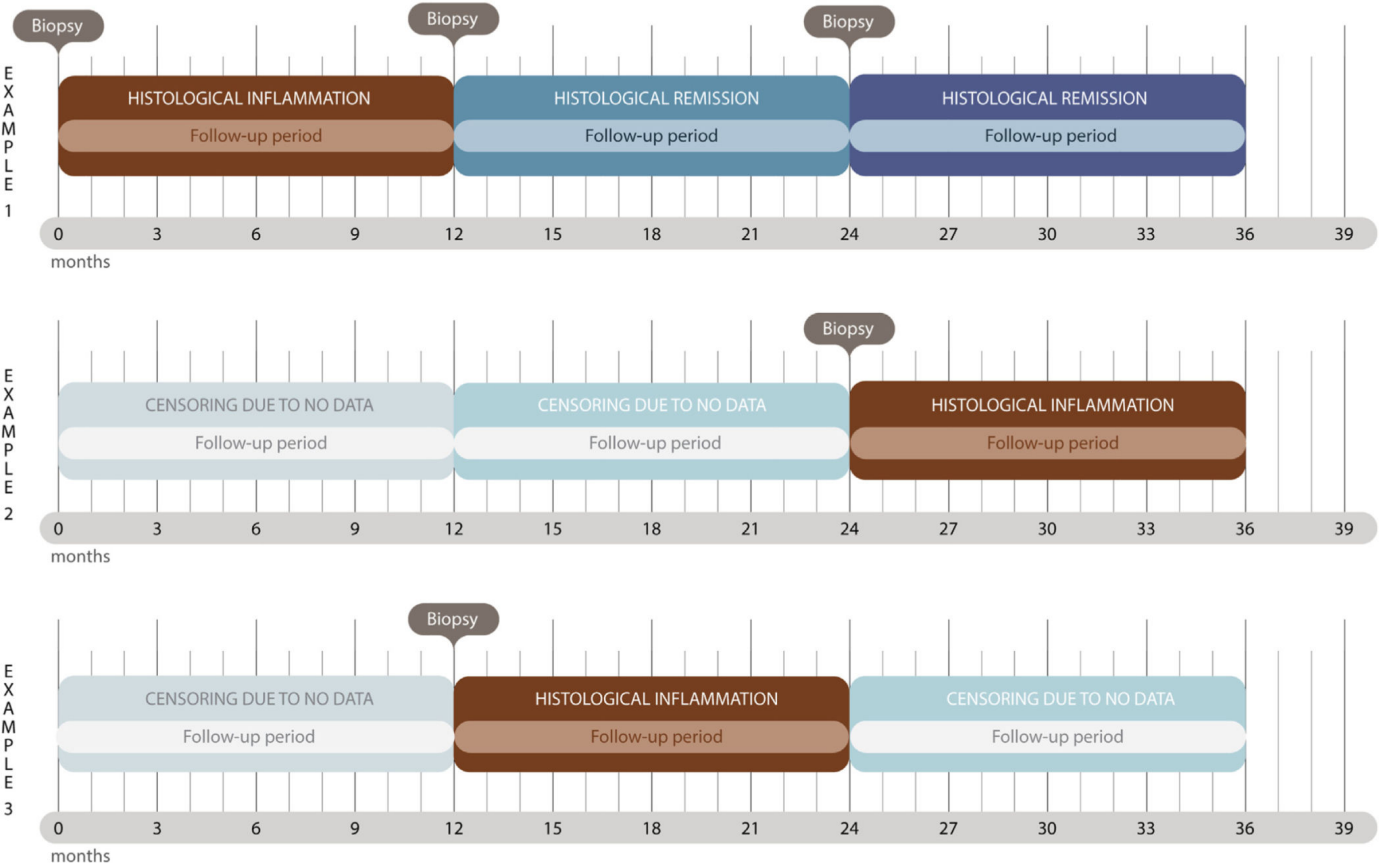
The figure exemplifies 3 individuals with 12-month exposure periods of histologic inflammation and histologic remission, as defined in Supplementary Table 7.

Although there is considerable interindividual variation in the disease course of IBD, we assumed that histologic inflammation and histologic remission, on average, would be reasonable predictors of histologic inflammation for up to 12 months (0–365 days) after biopsy (Figure 1). In the main analyses we therefore compared the risk of serious infections during the 12-month period after a biopsy showing histologic inflammation with the risk during the 12-month period after a biopsy showing histologic remission, whereas sensitivity analyses examined the risk of serious infections from 0 to <6 months after a biopsy. One individual with IBD could contribute to multiple inflammation and remission periods.

### Outcome: Serious Infections

Similar to previous works,^4,5^ we defined a serious infection as any inpatient infectious disease diagnosis, main or contributory, recorded in the NPR. Secondary analyses considered ear, nose, and throat/respiratory, gastrointestinal, musculoskeletal/skin, opportunistic, sepsis, and other infections, but did not consider surgical sites (Supplementary Table 6). Gastrointestinal infections may cause histologic inflammation with a risk of reverse causation. In a sensitivity analysis, we therefore defined any serious infections without considering gastrointestinal infections. We separately examined vaccine-preventable infections from influenza and hepatitis A (prior events of hepatitis B were excluded from our main analyses). Inpatient infectious disease data were retrieved from January 1, 1990, until December 31, 2016.

### Other Data

We gathered NPR data on selected chronic diseases (Supplementary Table 8), which may have impacted the relationship between IBD and serious infections. We used validated data from the NPR to identify any IBD-related surgery^15,16^ and hospitalizations as markers of disease severity (Supplementary Tables 2 and 3). We retrieved data from the LISA database on the highest attained education level as a proxy for socioeconomic status and the highest attained education level in parents for children (<18 years) with missing education information.^20^ Information on country of birth, age at index date (ie, the start of the exposure period), and calendar year were retrieved from the Swedish Total Population Register.^21^ Data were categorized as shown in Table 1.

### Medical Therapy

For analyses with follow-up evaluation since January 2006 and later, we examined the risk of any serious infection after accounting for medical IBD therapy that increased the risk of serious infections.^8^ Hence, we identified the current use of corticosteroids (systemic-local), immunomodulators (eg, thiopurines), or targeted therapies, including biologics. Current use was defined as dispensing/administration in the previous 6 months or fewer from the index date. Data were retrieved from 3 national registers: the NPR,^11^ the Swedish Prescribed Drug Register,^22^ and the Swedish Quality Register for IBD.^23,24^ Medications were identified using the Anatomical Therapeutic Chemical pharmaceutical classification system (Supplementary Table 9). The Prescribed Drug Register, established in July 2005 and in this study captured since January 1, 2006, includes prospectively recorded data on all dispensed prescriptions in Sweden.^22^

### Statistical Analyses

We used Cox regression models to estimate hazard ratios (HRs) for the time from biopsy to the first serious infection. Poisson regression was run to estimate incidence rate ratios (IRRs) of serious infections during histologic inflammation vs histologic remission to show the potential risk of repeated serious infections. Because the IRRs and HRs were virtually identical, we reported only HRs in the text. We estimated follow-up periods with vs without histologic inflammation starting from the biopsy date (Figure 1). Follow-up evaluation ended at the time of the first hospital admission for any serious infection or censoring after 12 months since biopsy, emigration, death, or end of data capture (December 31, 2016), whichever occurred first. In analyses of specific infectious disease categories, the time of outcome event was defined by the time of hospital admission for the particular infectious disease category. For individuals who underwent repeated biopsies, follow-up evaluation of histologic inflammation also ended if a second biopsy showing histologic remission was performed within 12 months from an earlier biopsy showing inflammation, and vice versa.

All analyses were adjusted for sex, age, calendar year, country of birth, education level, duration since IBD diagnosis, chronic comorbidities, any history of IBD-related surgery, or hospitalization.

#### Subanalyses.

We present HRs and IRRs by IBD subtypes (UC, CD, and IBD-unclassified), and the extent and location of the disease. Stratified analyses were performed by the following characteristics as defined by the start of each follow-up period: duration of IBD diagnosis (<2, ≥2 y), age (<18, 18–40, 40 to <50, 50 to <60, and ≥60 y), calendar year of IBD diagnosis (1990–1999, 2000–2009, and 2010–2016), education level (≤9, 10–12, and ≥13 y), and country of birth (Nordic, other).

#### Sensitivity analyses.

Analyses of the risk of any serious infection with follow-up evaluation since 2006 until 2016, in addition to the earlier-mentioned adjustment model, also were adjusted for the current (<6 months from the index date) use of medical IBD therapy, which may have increased the risk of serious infections. Because corticosteroid use or use of immunomodulators and targeted therapies may influence infection susceptibility,^8^ we also performed analyses stratified by any use of these drugs with fewer than 6 months from the index date. Moreover, we estimated the risk of serious infection during histologic inflammation (vs histologic remission) in individuals without clinically active IBD. For data restricted from 2006 to 2016, we defined clinical disease activity as in a recent report from our group,^18^ which was based on IBD-related surgery, hospitalization, budesonide or corticosteroid dispensing, initiation of immunomodulators, or targeted therapies.

Although our main analyses were based on individuals without a prior history of any inpatient infectious disease diagnosis (Supplementary Table 6), we performed sensitivity analyses without applying this exclusion criterion.

Finally, for follow-up periods from January 1, 2001, through 2016, we estimated the risk of any hospital-based infectious disease diagnosis, including inpatient and specialist outpatient care. Similar to our main analyses, our sensitivity analysis excluded individuals with any hospital-based (inpatient or outpatient) infectious disease diagnosis within 5 years from the index date.

## Results

Of 55,626 unique individuals with IBD, 43,523 individuals contributed to 68,666 exposure periods with histologic inflammation and 24,479 contributed to 34,680 exposure periods with histologic remission (because histologic inflammation was not required for IBD definition, which also could be defined based on repeated ICD codes for the disease, not all patients contributed to an exposure period of histologic inflammation). Some 12,376 (22%) individuals contributed to both 1 or more exposure periods of histologic inflammation and 1 or more exposure periods of histologic remission. Approximately two thirds of the individuals had UC and one third had CD (Table 1). The median number of biopsy reports per individual was 1 (range, 1–16 reports). Each report represented several microscopic analyses, but from 1 ileocolonoscopy.

Individuals with histologic inflammation had a lower education level and had, on average, a more recently diagnosed IBD (mean time since diagnosis, 4.4 y; SD, 5.5 y) than those with histologic remission (6.6 y; SD, 5.9 y) (Table 1). However, the sex distribution, age at diagnosis, and prevalence of chronic comorbidities were essentially similar across exposure groups (Table 1).

### Histologic Appearance and Risk of Serious Infections

**Figure 2.**
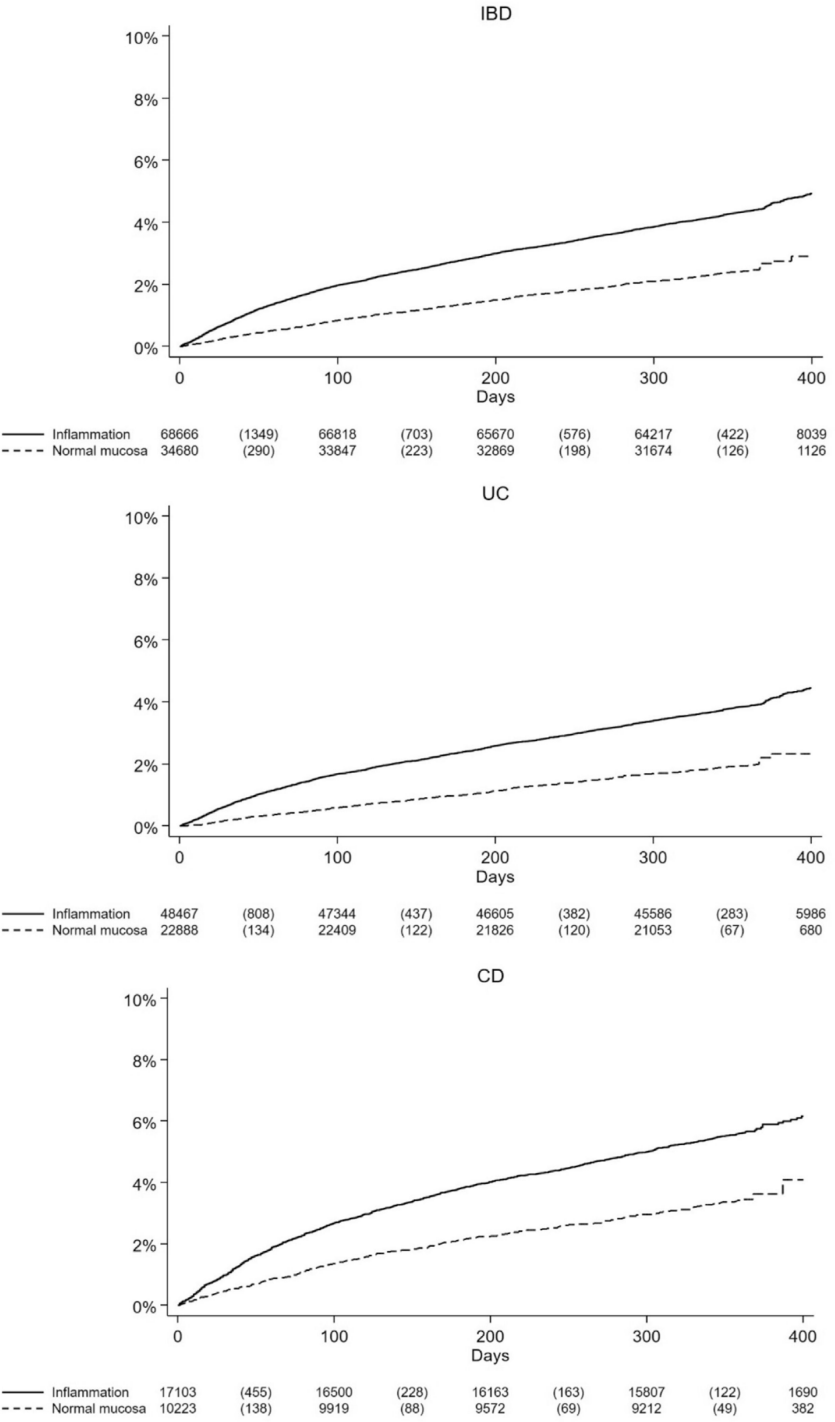
Kaplan–Meier curves of time to serious infection during 0 to <12 months of histologic inflammation vs histologic remission in inflammatory bowel disease (IBD), ulcerative colitis (UC), and Crohn’s disease (CD).

With vs without histologic inflammation, there were 4.62 (95% CI, 4.46–4.78) and 2.53 (95% CI, 2.36–2.70) serious infections per 100 person-years of follow-up evaluation, respectively, or 1 extra serious infection per 50 individuals with histologic inflammation followed up for 12 months. Accounting for sex, age, calendar year, disease duration, education level, country of birth, chronic comorbidities, IBD-related surgery, or hospitalization, histologic inflammation was associated with an adjusted HR (aHR) of 1.59 for serious infections (95% CI, 1.48–1.72) (Table 2). Figure 2 depicts the risk of serious infection over time by the histologic appearance of IBD.

Histologic inflammation was associated with an increased risk of serious infection in men (aHR, 1.40; 95% CI, 1.27–1.55) and women (aHR, 1.87; 95% CI, 1.66–2.11) in both UC (aHR, 1.68; 95% CI, 1.51–1.87) and CD (aHR, 1.59; 95% CI, 1.40–1.80). Although the aHRs of serious infection were higher in the 1990s than after 2010 (Table 2), they were otherwise relatively similar across age groups, education level, and duration of IBD diagnosis (Table 2).

### Categories of Serious Infections

Histologic inflammation (vs remission) in IBD was associated with an increased risk across categories of serious infections (Table 3). There were 0.43 (95% CI, 0.38–0.48) events of sepsis per 100 person-years of histologic inflammation compared with 0.23 (95% CI, 0.18–0.28) per 100 person-years of histologic remission (aHR, 1.66; 95% CI, 1.28–2.15). However, no significantly increased risk of influenza and hepatitis A were observed (Table 3), which Swedish IBD patients are vaccinated against routinely.

We found a 2-fold risk of sepsis (aHR, 1.95; 95% CI, 1.23–3.09) with histologic inflammation of CD and an aHR of 2.30 for opportunistic infections with histologic inflammation of UC (95% CI, 1.40–3.77) (Table 3).

### Sensitivity Analyses

After adjusting for the current use of medical IBD therapy, the presence of histologic inflammation (vs remission) was associated with an aHR of 1.47 (95% CI, 1.34–1.61) for serious infections (Table 4). Histologic inflammation (vs remission) was associated with an increased risk of serious infections in individuals with clinically quiescent IBD (aHR, 1.33; 95% CI, 1.13–1.57), and across strata of patients according to their use of corticosteroids or immunomodulators and targeted therapies (Table 4). Restricting the exposure period to the first 6 months after the biopsy (ie, 0 to <6 mo) yielded a somewhat higher aHR for serious infections of 1.72 (95% CI, 1.56–1.90) (Table 4).

Analyses of individuals with a history of an inpatient infectious disease diagnosis yielded broadly similar results as our main findings (aHR, 1.51; 95% CI, 1.42–1.61). Results were similar across strata of patients with fewer than 2 years or 2 years or longer disease duration, and restricting the outcome to serious infections being the main diagnoses also did not affect our estimates (Table 4).

## Discussion

In this nationwide study, histologic inflammation of IBD was associated with an increased risk of serious infections overall and across infectious disease categories, including sepsis. Our findings suggest that achieving histologic remission may reduce the risk of serious infections.

This nationwide IBD cohort, with histopathology data linked to national health registers, associates histologic inflammation of IBD with an increased risk of serious infections. Notably, the results remained robust after adjustment for medical IBD therapy. This finding is in line with emerging data suggesting that the largest risk factor for serious infections is IBD activity rather than targeted therapies.^9,25^ We also found consistently increased HRs across age groups. Although histologic inflammation has been linked more closely to clinical relapse in UC than CD,^26,27^ we found HRs of serious infections to be equally affected during histologic activity in UC and CD. Interestingly, aHRs of serious infection were higher in the 1990s than after 2010 (Table 2), suggesting that modern IBD care may have ameliorated the impact of histologic inflammation on infectious disease risk.

Compared with histologic remission, there was a 1.5- to 2-fold risk of sepsis and opportunistic infections in CD and UC with histologic inflammation. Both of these infection categories carry a high morbidity and mortality risk. The increased risk of sepsis was observed despite pneumococcal vaccination recommendations for IBD.^8^ Although most other effect sizes of this study were moderate in magnitude (the aHR for any serious infection was 1.59; 95% CI, 1.48–1.72), the findings are important given that infectious complications in IBD are common, often preventable, and convey an increased risk of death.

Potential biological explanations for the association between histologic inflammation of IBD and serious infections are disrupted intestinal permeability and malnutrition.^28^ In addition, low-grade inflammation through immune dysregulation may predispose to serious infections. In fact, high disease activity of rheumatoid arthritis has been linked to an increased risk of serious infection.^29^ Finally, the increased risk of serious infections with histologic inflammation also may be mediated through changes in the gut microbiome.^30^

### Strengths and Limitations

The major strength of this study was the use of population-based histopathology data as an objective measure of IBD activity. Our nationwide approach minimized selection bias, and our substantial power of more than 55,000 individuals with IBD who experienced more than 4000 serious infections enabled precise risk estimations. Our register-based definition of IBD has shown, on medical record review, a positive predictive value of 93% to 95% for a clinical diagnosis of IBD.^12,13^ Prospectively collected data, with independently retrieved outcome and exposure data, further reduce the risk of information bias. Multiple register linkages also allowed us to determine the association of histologic inflammation with serious infection independent of IBD-related surgery,^15^ hospital admission, and medical therapy.

We recognize that causal inference on observational data is fraught with difficulties. Residual confounding (ie, confounders missing or lacking in detail) may distort the results of any study in which practical or ethical considerations prevent randomized exposure allocation. For example, data were missing on smoking and body mass index, and we lacked details on the dosage of immuno-suppressive agents. A related limitation was our lack of data on endoscopic findings. There was also an absence of data on clinical symptoms and biomarkers and thus could not capture the clinical indications that may have prompted a histologic examination.

We expect a high validity of the infectious disease data from the NPR.^11^ Although the diagnostics of infectious diseases may have varied across counties and the study period, such differences should not be related to histologic activity and hence not cause spurious results. It also is unknown to what degree specific serious infections were laboratory-confirmed or diagnosed based on symptoms, signs, and epidemiologic data. Furthermore, we had no data on endoscopic or biochemical remission.

We acknowledge the possibility that routine histologic assessments may not capture the full burden of IBD activity, particularly in intramural, stricturing, or segmentally distributed intestinal inflammation of CD. Inflammation restricted to the ileum may be difficult to assess histologically. Medical management of IBD also may cause histologic activity to vary across colonic segments. In addition, in this nationwide study, misreading of individual microscopic analyses cannot be ruled out. The prospective nature of the study, where the exposure (histologic inflammation vs histologic remission) being recorded before the outcome (serious infection), ensures that the occurrence of a serious infection cannot have influenced histologic classification. Instead, misclassifying histopathologic data is more likely to attenuate the risk estimates. Our histology data were not based on a specific scoring system, and we could not examine whether less-than-complete histologic remission also may affect the risk of serious infection. We also lacked data on whether the histologic appearance changed over the 12-month period since the last biopsy. This lack of granularity in our data probably will attenuate the difference between histologic inflammation and histologic remission.

Finally, this study focused on the risk of serious infections requiring hospital admission. Consequently, our findings may not relate to milder infections in primary care settings. Our data originated from the universally accessible Swedish health care system, and the generalizability of results to other health care settings is unknown.

In conclusion, this nationwide IBD cohort study showed an increased risk of serious infections during histologic inflammation of IBD compared with when in histologic remission. This novel finding suggests that achieving histologic remission may diminish the risk of serious infection.

## Supplementary Material

1

## Figures and Tables

**Table 1.** Characteristics of Individuals With Histologic Inflammation and Histologic Remission of IBD in 1990 to 2016

	Biopsy in 1990–2016^a^	
Variable	Histologic inflammation	Histologic remission
Patients with IBD, N	43,523	24,479
Number of exposure periods per patient, N (%)		
1	28,849 (66.3)	17,734 (72.4)
2	8789 (20.2)	4550 (18.6)
3	3352 (7.7)	1397 (5.7)
≥4	2533 (5.8)	798 (3.3)
**Exposure periods, N**	68,666^b^	34,680^b^
Sex, n (%)		
Males	37,284 (54.3)	18,099 (52.2)
Females	31,382 (45.7)	16,581 (47.8)
Year of index date,^c^ n (%)		
1990–1999	11,391 (16.6)	2099 (6.1)
2000–2009	29,779 (43.4)	13,548 (39.1)
2010–2016	27,496 (40.0)	19,033 (54.9)
Age at index date,^c^ *y*		
Mean (SD)	43.5 (18.0)	45.3 (16.9)
Categories, n (%)		
<18 y	4795 (7.0)	1871 (5.4)
18 to <40 y	26,282 (38.3)	11,730 (33.8)
40 to <50 y	23,306 (33.9)	13,448 (38.8)
50 to <60 y	14,283 (20.8)	7631 (22.0)
≥60 y Year of IBD diagnosis,^d^ n (%)	4795 (7.0)	1871 (5.4)
1990–1999	24,543 (35.7)	10,521 (30.3)
2000–2009	32,049 (46.7)	18,959 (54.7)
2010–2016	12,074 (17.6)	5200 (15.0)
Age at IBD diagnosis,^d^ *y*		
Mean (SD)	39.2 (17.8)	38.7 (16.5)
Categories, n (%)		
<18 y	7943 (11.6)	4023 (11.6)
18 to <40 y	29,620 (43.1)	14,648 (42.2)
40 to <50 y	21,157 (30.8)	12,174 (35.1)
50 to <60 y	9946 (14.5)	3835 (11.1)
≥60 y	7943 (11.6)	4023 (11.6)
Level of education,^e^ n (%)		
≤9 y	16,282 (23.7)	6866 (19.8)
10–12 y	31,997 (46.6)	15,827 (45.6)
≥13 y	20,201 (29.4)	11,944 (34.4)
Missing	186 (0.3)	43 (0.1)
Country of birth, n (%)		
Nordic	64,277 (93.6)	32,294 (93.1)
Non-Nordic	3923 (5.7)	2129 (6.1)
Missing	466 (0.7)	257 (0.7)
Disease duration, *y* Mean (SD)	4.4 (5.5)	6.6 (5.9)
Categories, n (%)		
<2 y	34,927 (50.9)	10,651 (30.7)
≥2 y	33,739 (49.1)	24,029 (69.3)
IBD type, n (%)		
CD	17,103 (24.9)	10,223 (29.5)
UC	48,467 (70.6)	22,888 (66.0)
IBD-U	3096 (4.5)	1569 (4.5)
Montreal classification CD,^f^ n (%) L1/L3/LX	11,146 (65.2)	7132 (69.8)
L2	3996 (23.4)	2493 (24.4)
L missing/ICD code before 1997	1961 (11.5)	598 (5.8)
Montreal classification UC,^f^ n (%)		
E1/E2	12,794 (26.4)	6292 (27.5)
E3	18,472 (38.1)	10,586 (46.3)
EX	11,171 (23.0)	4744 (20.7)
E missing/ICD code before 1997	6030 (12.4)	1266 (5.5)
Health care and drug use Any hospitalization from 24 to 6 months before the index date^c^		
Mean (SD)	3.1 (4.8)	3.9 (5.3)
IBD-related hospitalization,^g^ n (%)	39,539 (57.6)	19,609 (56.5)
IBD-related surgery,^g^ n (%)	12,025 (17.5)	4753 (13.7)
IBD-related drug use <6 months before index date,^h^ n (%)	16,739 (41.1)	8897 (34.2)
Corticosteroid	11,313 (27.8)	4435 (17.1)
Budesonide	2399 (5.9)	1336 (5.1)
Thiopurines	5908 (14.5)	4230 (16.3)
Targeted therapy in IBD	1979 (4.9)	1122 (4.3)
Chronic comorbidity, n (%)^i^		
Diabetes	2458 (3.6)	1362 (3.9)
Hypertension	4765 (6.9)	2918 (8.4)
Asthma	2610 (3.8)	1636 (4.7)
Autoimmune disease	4045 (5.9)	2515 (7.3)
Follow-up time, *d* Mean (SD)	377 (115)	355 (81)
Median (IQR)	365 (365–365)	365 (365–365)

CD, Crohn’s disease; IBD, inflammatory bowel disease; IBD-U, inflammatory bowel disease–unclassified; ICD, International Classification of Diseases; IQR, interquartile range; UC, ulcerative colitis.

a Histopathology reports, as recorded in the Epidemiology Strengthened by histoPathology Reports in Sweden cohort,^10^ from the ileocolorectum showing inflammation and normal histology, as detailed in Supplementary Table 7.

b Data presented by exposure period. The expopsure periods correspond to the following number of unique individuals: histologic inflammation, n = 43,523; and histologic remission, n = 24,479.

c The index date equals the start of the exposure period.

d The time of IBD diagnosis was defined as the time of the second (out of minimum 2) diagnostic listings for IBD or related histopathology (Systematized Nomenclature of Medicine) code.

e In children with missing data on education level, we used the highest attained education level of the parent.

f Extent and location of disease at the time of diagnosis as detailed in Supplementary Table 2.

g Any time before the index date. IBD-related hospitalization equaled an inpatient visit with a main diagnosis for IBD. IBD-related surgery was defined by relevant surgical codes listed in Supplementary Table 3.

h Medical IBD therapy is defined in Supplementary Table 9.

i Any time before the index date as detailed in Supplementary Table 8.

**Table 2.** Risk of Serious Infection During 0 to <12 Months of Histologic Inflammation and Histologic Remission of IBD

	N (% of total)		Infections, N (%)		Follow-up period, *y*		IR (95% CI) by 100 PY			
	Histologic inflammation	Histologic remission	Histologic inflammation	Histologic remission	Histologic inflammation	Histologic remission	Histologic inflammation	Histologic remission	IRR^a^ (95% CI)	HR^a^ (95% CI)
Overall	68,666 (100)	34,680 (100)	3271 (4.8)	853 (2.5)	70,846	33,666	4.62 (4.46–4.78)	2.53 (2.36–2.70)	1.60 (1.48–1.73)	1.59 (1.48–1.72)
Sex										
Males	37,284 (54.3)	18,099 (52.2)	1790 (4.8)	502 (2.8)	38,679	17,513	4.63 (4.41–4.84)	2.87 (2.62–3.12)	1.41 (1.27–1.56)	1.40 (1.27–1.55)
Females	31,382 (45.7)	16,581 (47.8)	1481 (4.7)	351 (2.1)	32,167	16,152	4.60 (4.37–4.84)	2.17 (1.95–2.40)	1.88 (1.67–2.12)	1.87 (1.66–2.11)
Index date,^b^ *y*										
1990–1999	11,391 (16.6)	2099 (6.1)	677 (5.9)	55 (2.6)	12,175	2048	5.56 (5.14–5.98)	2.69 (1.98–3.39)	1.88 (1.42–2.47)	1.92 (1.46–2.53)
2000–2009	29,779 (43.4)	13,548 (39.1)	1379 (4.6)	343 (2.5)	31,032	13,459	4.44 (4.21–4.68)	2.55 (2.28–2.82)	1.60 (1.42–1.80)	1.59 (1.41–1.79)
2010–2016	27,496 (40.0)	19,033 (54.9)	1215 (4.4)	455 (2.4)	27,639	18,158	4.40 (4.15–4.64)	2.51 (2.28–2.74)	1.51 (1.35–1.68)	1.49 (1.34–1.67)
Disease duration, *y*										
<2 y	34,927 (50.9)	10,651 (30.7)	2007 (5.7)	362 (3.4)	36,226	10,382	5.54 (5.30–5.78)	3.49 (3.13–3.85)	1.54 (1.38–1.73)	1.55 (1.38–1.74)
≥2 y	33,739 (49.1)	24,029 (69.3)	1264 (3.7)	491 (2.0)	34,620	23,283	3.65 (3.45–3.85)	2.11 (1.92–2.30)	1.58 (1.42–1.76)	1.55 (1.40–1.73)
Age at index date^b^										
<18 y	4795 (7.0)	1871 (5.4)	247 (5.2)	60 (3.2)	5052	1817	4.89 (4.28–5.50)	3.30 (2.47–4.14)	1.46 (1.10–1.94)	1.44 (1.08–1.92)
18 to <40 y	26,282 (38.3)	11,730 (33.8)	1115 (4.2)	289 (2.5)	27,319	11,376	4.08 (3.84–4.32)	2.54 (2.25–2.83)	1.48 (1.29–1.68)	1.47 (1.28–1.68)
40 to <50 y	23,306 (33.9)	13,448 (38.8)	833 (3.6)	231 (1.7)	24,220	13,107	3.44 (3.21–3.67)	1.76 (1.54–1.99)	1.67 (1.44–1.94)	1.66 (1.43–1.93)
50 to <60 y	14,283 (20.8)	7631 (22.0)	1076 (7.5)	273 (3.6)	14,254	7364	7.55 (7.10–8.00)	3.71 (3.27–4.15)	1.51 (1.31–1.73)	1.51 (1.32–1.74)
≥60 y	4795 (7.0)	1871 (5.4)	247 (5.2)	60 (3.2)	5052	1817	4.89 (4.28–5.50)	3.30 (2.47–4.14)	1.46 (1.10–1.94)	1.44 (1.08–1.92)
Level of education										
≤9 y	16,282 (23.7)	6866 (19.8)	1089 (6.7)	239 (3.5)	16,695	6672	6.52 (6.14–6.91)	3.58 (3.13–4.04)	1.64 (1.42–1.89)	1.51 (1.32–1.74)
10–12 y	31,997 (46.6)	15,827 (45.6)	1448 (4.5)	385 (2.4)	33,134	15,369	4.37 (4.14–4.60)	2.51 (2.25–2.76)	1.59 (1.41–1.78)	1.51 (1.32–1.74)
≥13 y	20,201 (29.4)	11,944 (34.4)	706 (3.5)	227 (1.9)	20,852	11,577	3.39 (3.14–3.64)	1.96 (1.71–2.22)	1.53 (1.31–1.78)	1.51 (1.32–1.74)
Country of birth, n (%)										
Nordic	64,277 (93.6)	32,294 (93.1)	3077 (4.8)	791 (2.4)	66,324	31,390	4.64 (4.48–4.80)	2.52 (2.34–2.70)	1.62 (1.49–1.75)	1.61 (1.49–1.74)
Non-Nordic	3923 (5.7)	2129 (6.1)	180 (4.6)	56 (2.6)	4034	2029	4.46 (3.81–5.11)	2.76 (2.04–3.48)	1.39 (1.03–1.89)	1.40 (1.03–1.90)
IBD type										
CD	17,103 (24.9)	10,223 (29.5)	1015 (5.9)	352 (3.4)	17,259	9858	5.88 (5.52–6.24)	3.57 (3.20–3.94)	1.58 (1.40–1.79)	1.59 (1.40–1.80)
UC	48,467 (70.6)	22,888 (66.0)	2070 (4.3)	450 (2.0)	50,453	22,297	4.10 (3.93–4.28)	2.02 (1.83–2.20)	1.69 (1.52–1.88)	1.68 (1.51–1.87)
IBD-U	3096 (4.5)	1569 (4.5)	186 (6.0)	51 (3.3)	3134	1509	5.93 (5.08–6.79)	3.38 (2.45–4.30)	1.50 (1.09–2.06)	1.48 (1.08–2.04)
Montreal classification CD^c^										
L1/L3/LX	11,146 (73.6)	7132 (74.1)	696 (6.2)	274 (3.8)	11,127	6858	6.25 (5.79–6.72)	4.00 (3.52–4.47)	1.53 (1.32–1.76)	1.53 (1.32–1.76)
L2	3996 (26.4)	2493 (25.9)	213 (5.3)	67 (2.7)	4069	2411	5.23 (4.53–5.94)	2.78 (2.11–3.44)	1.75 (1.32–2.33)	1.74 (1.31–2.32)
Montreal classification UC^C^										
E1/E2	12,794 (30.1)	6292 (29.1)	443 (3.5)	94 (1.5)	13,192	6123	3.36 (3.05–3.67)	1.54 (1.22–1.85)	1.97 (1.57–2.47)	1.91 (1.52–2.40)
E3	18,472 (43.5)	10,586 (49.0)	820 (4.4)	240 (2.3)	19,083	10,262	4.30 (4.00–59)	2.34 (2.04–2.63)	1.46 (1.26–1.70)	1.45 (1.25–1.68)
EX	11,171 (26.3)	4744 (21.9)	474 (4.2)	95 (2.0)	11,679	4653	4.06 (3.69–42)	2.04 (1.63–2.45)	1.73 (1.38–2.17)	1.75 (1.39–2.19)

NOTE. Histologic inflammation and histologic remission as defined by ileocolorectal histology codes listed in Supplementary Table 7.

CD, Crohn’s disease; HR, hazard ratio; IR, incidence rate; IRR, incidence rate ratio; IBD, inflammatory bowel disease; IBD-U, inflammatory bowel disease–unclassified; PY, person-years; UC, ulcerative colitis.

a Adjusted for age at index date (ie, start of exposure period), sex, calendar year, education level, country of birth, disease duration, any history of inpatient IBD care, IBD-related surgery, and chronic comorbidity (diabetes, hypertension, chronic autoimmune disease, and asthma) (Supplementary Table 8).

b Index date equals the start of the exposure period.

c Extent and location of disease at diagnosis as detailed in Supplementary Table 2.

**Table 3.** Risk Serious Infection Categories During 0 to <12 Months of Histologic Inflammation and Histologic Remission of IBD

	Infections, N (%)		Follow-up period, *y*		IR (95% CI) by 100 PY			
	Histologic inflammation	Histologic remission	Histologic inflammation	Histologic remission	Histologic inflammation	Histologic remission	IRR^a^ (95% CI)	HR^a^ (95% CI)
IBD overall								
Any infection^b^	3271 (4.8)	853 (2.5)	70,846	33,666	4.62 (4.46–4.78)	2.53 (2.36–2.70)	1.60 (1.48–1.73)	1.59 (1.48–1.72)
Sepsis	315 (0.5)	77 (0.2)	73,005	34,088	0.43 (0.38–0.48)	0.23 (0.18–0.28)	1.66 (1.29–2.15)	1.66 (1.28–2.15)
ENT/respiratory	812 (1.2)	225 (0.6)	72,688	34,009	1.12 (1.04–1.19)	0.66 (0.58–0.75)	1.48 (1.27–1.72)	1.47 (1.26–1.71)
Gastrointestinal	1210 (1.8)	278 (0.8)	72,320	33,975	1.67 (1.58–1.77)	0.82 (0.72–0.91)	1.71 (1.50–1.96)	1.72 (1.50–1.96)
Musculoskeletal/skin	282 (0.4)	69 (0.2)	73,030	34,083	0.39 (0.34–0.43)	0.20 (0.15–0.25)	1.81 (1.39–2.37)	1.78 (1.36–2.33)
Opportunistic	177 (0.3)	44 (0.1)	73,085	34,102	0.24 (0.21–0.28)	0.13 (0.09–0.17)	1.75 (1.25–2.45)	1.71 (1.22–2.41)
Other	1169 (1.7)	321 (0.9)	72,362	33,947	1.62 (1.52–1.71)	0.95 (0.84–1.05)	1.53 (1.35–1.74)	1.51 (1.33–1.72)
Influenza or hepatitis A^c^	39 (0.1)	10 (0.0)	73,189	34,117	0.05 (0.04–0.07)	0.03 (0.01–0.05)	1.66 (0.81–3.38)	1.62 (0.79–3.32)
CD								
Any infection^b^	1015 (5.9)	352 (3.4)	17,259	9858	5.88 (5.52–6.24)	3.57 (3.20–3.94)	1.58 (1.40–1.79)	1.59 (1.40–1.80)
Sepsis	90 (0.5)	24 (0.2)	17,939	10,049	0.50 (0.40–0.61)	0.24 (0.14–0.33)	1.92 (1.21–3.05)	1.95 (1.23–3.09)
ENT/respiratory	216 (1.3)	85 (0.8)	17,862	10,016	1.21 (1.05–1.37)	0.85 (0.67–1.03)	1.40 (1.08–1.82)	1.42 (1.09–1.84)
Gastrointestinal	408 (2.4)	134 (1.3)	17,680	9982	2.31 (2.08–2.53)	1.34 (1.12–1.57)	1.55 (1.27–1.89)	1.56 (1.28–1.91)
Musculoskeletal/skin	82 (0.5)	26 (0.3)	17,947	10,043	0.46 (0.36–0.56)	0.26 (0.16–0.36)	1.89 (1.20–2.96)	1.87 (1.19–2.93)
Opportunistic	52 (0.3)	24 (0.2)	17,964	10,049	0.29 (0.21–0.37)	0.24 (0.14–0.33)	1.31 (0.80–2.16)	1.32 (0.80–2.17)
Other	346 (2.0)	122 (1.2)	17,745	9990	1.95 (1.74–2.16)	1.22 (1.00–1.44)	1.61 (1.30–1.98)	1.59 (1.28–1.96)
Influenza or hepatitis A^c^	11 (0.1)	5 (0.0)	17,996	10,056	0.06 (0.03–0.10)	0.05 (0.01–0.09)	1.36 (0.46–4.03)	1.36 (0.46–4.05)
UC								
Any infection^b^	2070 (4.3)	450 (2.0)	50,453	22,297	4.10 (3.93–4.28)	2.02 (1.83–2.20)	1.69 (1.52–1.88)	1.68 (1.51–1.87)
Sepsis	209 (0.4)	50 (0.2)	51,811	22,502	0.40 (0.35–0.46)	0.22 (0.16–0.28)	1.55 (1.12–2.13)	1.53 (1.11–2.11)
ENT/respiratory	542 (1.1)	119 (0.5)	51,593	22,467	1.05 (0.96–1.14)	0.53 (0.43–0.62)	1.66 (1.36–2.04)	1.64 (1.33–2.01)
Gastrointestinal	724 (1.5)	128 (0.6)	51,433	22,461	1.41 (1.31–1.51)	0.57 (0.47–0.67)	1.93 (1.59–2.34)	1.92 (1.59–2.33)
Musculoskeletal/skin	181 (0.4)	40 (0.2)	51,833	22,504	0.35 (0.30–0.40)	0.18 (0.12–0.23)	1.73 (1.22–2.46)	1.69 (1.19–2.41)
Opportunistic	120 (0.2)	19 (0.1)	51,861	22,516	0.23 (0.19–0.27)	0.08 (0.05–0.12)	2.38 (1.45–3.90)	2.30 (1.40–3.77)
Other	764 (1.6)	180 (0.8)	51,398	22,430	1.49 (1.38–1.59)	0.80 (0.69–0.92)	1.57 (1.32–1.85)	1.55 (1.31–1.83)
Influenza or hepatitis A^c^	28 (0.1)	5 (0.0)	51,930	22,522	0.05 (0.03–0.07)	0.02 (0.00–0.04)	1.91 (0.72–5.06)	1.85 (0.69–4.94)

NOTE. Histologic inflammation and histologic remission as defined by ileocolorectal histology codes listed in Supplementary Table 7.

CD, Crohn’s disease; ENT, ear, nose, and throat; HR, hazard ratio; IBD, inflammatory bowel disease; IR, incidence rate; IRR, incidence rate ratio; PY, person-years; UC, ulcerative colitis.

a Adjusted for age at index date (ie, start of exposure period), sex, calendar year, education level, country of birth, disease duration, any history of inpatient IBD care, IBD-related surgery, and chronic comorbidity (Supplementary Table 8).

b Serious infection categories as detailed in Supplementary Table 6.

c Influenza is included in the category of “ENT/respiratory.” Hepatitis A is included in the category of “other.”

**Table 4.** Sensitivity Analyses for the Risk of Serious Infection (Inpatient Diagnosis) and Hospital-Based Infections (Inpatient or Outpatient Diagnosis) During 0 to < 12 Months of Histologic Inflammation and Histologic Remission of IBD

	N (% of total)		Infections, N (%)		Follow-up period, *y*		IR (95% CI) by 100 PY			
	Histologic inflammation	Histologic remission	Histologic inflammation	Histologic remission	Histologic inflammation	Histologic remission	Histologic inflammation	Histologic remission	IRR^a^ (95% CI)	HR^a^ (95% CI)
Serious infections, years 1990–2016										
Main analyses (for reference)										
IBD overall^b^	68,666 (100)	34,680 (100)	3271 (4.8)	853 (2.5)	70,846	33,666	4.62 (4.46–4.78)	2.53 (2.36–2.70)	1.60 (1.48–1.73)	1.59 (1.48–1.72)
CD	17,103 (24.9)	10,223 (29.5)	1015 (5.9)	352 (3.4)	17,259	9858	5.88 (5.52–6.24)	3.57 (3.20–3.94)	1.58 (1.40–1.79)	1.59 (1.40–1.80)
UC	48,467 (70.6)	22,888 (66.0)	2070 (4.3)	450 (2.0)	50,453	22,297	4.10 (3.93–4.28)	2.02 (1.83–2.20)	1.69 (1.52–1.88)	1.68 (1.51–1.87)
Not excluding individuals with prior serious infection										
IBD overall^b^	79,242 (100)	38,859 (100)	4925 (6.2)	1345 (3.5)	80,930	37,517	6.09 (5.92–6.26)	3.58 (3.39–3.78)	1.51 (1.42–1.60)	1.51 (1.42–1.61)
CD	20,713 (26.1)	12,024 (30.9)	1585 (7.7)	596 (5.0)	20,657	11,499	7.67 (7.29–8.05)	5.18 (4.77–5.60)	1.43 (1.30–1.58)	1.44 (1.31–1.59)
UC	54,780 (69.1)	25,027 (64.4)	3017 (5.5)	668 (2.7)	56,555	24,288	5.33 (5.14–5.52)	2.75 (2.54–2.96)	1.62 (1.48–1.76)	1.62 (1.48–1.76)
Only main diagnosis										
IBD overall	68,666 (100)	34,680 (100)	2071 (3.0)	584 (1.7)	71,788	33,805	2.88 (2.76–3.01)	1.73 (1.59–1.87)	1.49 (1.36–1.64)	1.48 (1.35–1.63)
CD	17,103 (24.9)	10,223 (29.5)	632 (3.7)	240 (2.3)	17,561	9919	3.60 (3.32–3.88)	2.42 (2.11–2.73)	1.46 (1.25–1.70)	1.45 (1.24–1.69)
UC	48,467 (70.6)	22,888 (66.0)	1316 (2.7)	302 (1.3)	51,048	22,369	2.58 (2.44–2.72)	1.35 (1.20–1.50)	1.63 (1.43–1.86)	1.62 (1.42–1.84)
Any serious infection with gastrointestinal infection excluded										
IBD overall	68,666 (100)	34,680 (100)	2314 (3.4)	625 (1.8)	71,576	33,795	3.23 (3.10–3.36)	1.85 (1.70–1.99)	1.57 (1.43–1.72)	1.55 (1.41–1.70)
CD	17,103 (24.9)	10,223 (29.5)	670 (3.9)	241 (2.4)	17,535	9925	3.82 (3.53–4.11)	2.43 (2.12–2.73)	1.58 (1.36–1.84)	1.57 (1.35–1.82)
UC	48,467 (70.6)	22,888 (66.0)	1513 (3.1)	344 (1.5)	50,866	22,354	2.97 (2.82–3.12)	1.54 (1.38–1.70)	1.65 (1.46–1.86)	1.62 (1.44–1.83)
Within 0–6 months after histologic inflammation vs remission										
IBD overall	73,514 (100)	36,905 (100)	2203 (3.0)	528 (1.4)	37,517	18,146	5.87 (5.63–6.12)	2.91 (2.66–3.16)	1.72 (1.56–1.90)	1.72 (1.56–1.90)
CD	18,128 (24.7)	10,899 (29.5)	715 (3.9)	240 (2.2)	9110	5343	7.85 (7.27–8.42)	4.49 (3.92–5.06)	1.66 (1.43–1.92)	1.66 (1.43–1.93)
UC	52,074 (70.8)	24,326 (65.9)	1357 (2.6)	260 (1.1)	26,735	11,977	5.08 (4.81–5.35)	2.17 (1.91–2.43)	1.88 (1.64–2.15)	1.87 (1.63–2.15)
Disease duration, *y*										
IBD overall										
<2 y	34,927 (50.9)	10,651 (30.7)	2007 (5.7)	362 (3.4)	36,226	10,382	5.54 (5.30–5.78)	3.49 (3.13–3.85)	1.54 (1.38–1.73)	1.55 (1.38–1.74)
≥2 y	33,739 (49.1)	24,029 (69.3)	1264 (3.7)	491 (2.0)	34,620	23,283	3.65 (3.45–3.85)	2.11 (1.92–2.30)	1.58 (1.42–1.76)	1.55 (1.40–1.73)
CD										
<2 y	9117 (53.3)	3607 (35.3)	635 (7.0)	164 (4.5)	9204	3514	6.90 (6.36–7.44)	4.67 (3.95–5.38)	1.54 (1.29–1.83)	1.55 (1.30–1.84)
≥2 y	7986 (46.7)	6616 (64.7)	380 (4.8)	188 (2.8)	8054	6343	4.72 (4.24–5.19)	2.96 (2.54–3.39)	1.55 (1.30–1.86)	1.55 (1.29–1.85)
UC										
<2 y	23,865 (49.2)	6343 (27.7)	1227 (5.1)	170 (2.7)	25,063	6194	4.90 (4.62–5.17)	2.74 (2.33–3.16)	1.63 (1.39–1.92)	1.64 (1.39–1.93)
≥2 y	24,602 (50.8)	16,545 (72.3)	843 (3.4)	280 (1.7)	25,389	16,103	3.32 (3.10–3.54)	1.74 (1.54–1.94)	1.68 (1.47–1.93)	1.65 (1.43–1.89)
Serious infections, years 2006–2016^c^										
Adjustment for medical IBD therapy										
IBD overall^b^	40,745 (100)	25,994 (100)	1839 (4.5)	628 (2.4)	41,425	25,078	4.44 (4.24–4.64)	2.50 (2.31–2.70)	1.48 (1.35–1.62)	1.47 (1.34–1.61)
CD	10,198 (25.0)	7629 (29.3)	604 (5.9)	262 (3.4)	10,166	7299	5.94 (5.47–6.42)	3.59 (3.15–4.02)	1.48 (1.28–1.72)	1.48 (1.27–1.72)
UC	28,265 (69.4)	17,079 (65.7)	1099 (3.9)	323 (1.9)	28,983	16,540	3.79 (3.57–4.02)	1.95 (1.74–2.17)	1.55 (1.36–1.76)	1.53 (1.35–1.74)
Clinically quiescent IBD^d^										
IBD overall	36,428 (100)	23,582 (100)	444 (1.2)	216 (0.9)	25,141	17,915	1.77 (1.60–1.93)	1.21 (1.04–1.37)	1.34 (1.14–1.59)	1.33 (1.13–1.57)
CD	7942 (21.8)	6485 (27.5)	117 (1.5)	73 (1.1)	4771	4444	2.45 (2.01–2.90)	1.64 (1.27–2.02)	1.50 (1.12–2.03)	1.49 (1.11–2.01)
UC	26,491 (72.7)	15,916 (67.5)	295 (1.1)	124 (0.8)	19,017	12,593	1.55 (1.37–1.73)	0.98 (0.81–1.16)	1.37 (1.10–1.70)	1.36 (1.09–1.69)
Corticosteroid use <6 months from index date										
IBD overall	11,313 (100)	4435 (100)	683 (6.0)	181 (4.1)	11,664	4245	5.86 (5.42–6.29)	4.26 (3.64–4.88)	1.35 (1.14–1.59)	1.34 (1.13–1.58)
CD	2808 (24.8)	1372 (30.9)	208 (7.4)	72 (5.2)	2826	1308	7.36 (6.36–8.36)	5.50 (4.23–6.77)	1.40 (1.07–1.84)	1.39 (1.06–1.83)
UC	7845 (69.3)	2800 (63.1)	431 (5.5)	97 (3.5)	8164	2687	5.28 (4.78–5.78)	3.61 (2.89–4.33)	1.42 (1.13–1.77)	1.40 (1.12–1.75)
No corticosteroid use < 6 months from index date										
IBD overall	29,432 (100)	21,559 (100)	1 156 (3.9)	447 (2.1)	29,760	20,833	3.88 (3.66–4.11)	2.15 (1.95–2.34)	1.51 (1.35–1.69)	1.50 (1.34–1.68)
CD	7390 (25.1)	6257 (29.0)	396 (5.4)	190 (3.0)	7340	5991	5.40 (4.86–5.93)	3.17 (2.72–3.62)	1.52 (1.28–1.82)	1.52 (1.28–1.82)
UC	20,420 (69.4)	14,279 (66.2)	668 (3.3)	226 (1.6)	20,819	13,853	3.21 (2.97–3.45)	1.63 (1.42–1.84)	1.60 (1.37–1.86)	1.57 (1.34–1.84)
Immunomodulator or targeted therapy use < 6 months from index date										
IBD overall	7111 (100)	4958 (100)	348 (4.9)	140 (2.8)	7260	4726	4.79 (4.29–5.30)	2.96 (2.47–3.45)	1.46 (1.20–1.79)	1.46 (1.19–1.78)
CD	2647 (37.2)	2009 (40.5)	151 (5.7)	65 (3.2)	2660	1917	5.68 (4.77–6.58)	3.39 (2.57–4.21)	1.56 (1.16–2.10)	1.55 (1.15–2.08)
UC	4184 (58.8)	2739 (55.2)	184 (4.4)	65 (2.4)	4317	2609	4.26 (3.65–4.88)	2.49 (1.89–3.10)	1.50 (1.12–1.99)	1.49 (1.12–1.99)
No immunomodulator or targeted therapy use <6 months from index date										
IBD overall	33,634 (100)	21,036 (100)	1491 (4.4)	488 (2.3)	34,164	20,352	4.36 (4.14–4.59)	2.40 (2.19–2.61)	1.55 (1.40–1.72)	1.54 (1.38–1.71)
CD	7551 (22.5)	5620 (26.7)	453 (6.0)	197 (3.5)	7505	5382	6.04 (5.48–6.59)	3.66 (3.15–4.17)	1.48 (1.25–1.76)	1.49 (1.25–1.76)
UC	24,081 (71.6)	14,340 (68.2)	915 (3.8)	258 (1.8)	24,666	13,930	3.71 (3.47–3.95)	1.85 (1.63–2.08)	1.67 (1.45–1.93)	1.65 (1.43–1.90)
Hospital-based infection, years 2001–2016^e^										
Hospital-based infection										
IBDoverall^b^	47,790 (100)	27,559 (100)	3703 (7.7)	1489 (5.4)	47,938	26,319	7.72 (7.48–7.97)	5.66 (5.37–5.94)	1.24 (1.16–1.31)	1.24 (1.16–1.31)
CD	11,402 (23.9)	7744 (28.1)	1146 (10.1)	546 (7.1)	11,112	7300	10.31 (9.72–10.91)	7.48 (6.85–8.11)	1.28 (1.16–1.42)	1.28 (1.16–1.42)
UC	34,146 (71.5)	18,580 (67.4)	2345 (6.9)	870 (4.7)	34,618	17,840	6.77 (6.50–7.05)	4.88 (4.55–5.20)	1.24 (1.14–1.34)	1.23 (1.14–1.33)

NOTE. Histologic inflammation and histologic remission as defined by ileocolorectal histology codes listed in Supplementary Table 7.

CD, Crohn’s disease; HR, hazard ratio; IBD, inflammatory bowel disease; IR, incidence rate; IRR, incidence rate ratio; PY, person-years; UC, ulcerative colitis.

a Adjusted for age at index date (ie, start of exposure period), sex, calendar year, education level, country of birth, disease duration, any history of inpatient IBD care, IBD-related surgery, and chronic comorbidity (Supplementary Table 8).

b Any IBD subtype including IBD-unclassified, which, because of few events, was not examined separately in these sensitivity analyses.

c Follow-up evaluation since 2006. Additional adjustment for current use of medical IBD therapy as detailed in Supplementary Table 9.

d Clinical disease activity as in a recent report from our group,^18^ and based on IBD-related surgery, hospitalization, budesonide or corticosteroid dispensing, initiation of immunomodulators, or targeted therapies.

e Follow-up evaluation since 2001. Excluding individuals with any inpatient or outpatient infectious disease diagnosis within 5 years of index date.

## Data Availability

No additional data are available due to Swedish regulations.

## What You Need to Know

### Background

Inflammatory bowel disease (IBD) has been linked to an increased risk of serious infections, but it is unclear how this risk varies by histologic activity.

### Findings

Compared with IBD patients in remission, IBD patients with histologic inflammation had an increased risk of serious infections overall and across infectious disease categories, including sepsis.

### Implications for patient care

Our findings suggest that achieving histologic remission of IBD may reduce the risk of serious infections.

## Abbreviations used in this paper:

aHR: adjusted hazard ratio
CD: Crohn’s disease
HR: hazard ratio
IRR: incidence rate ratio
IBD: inflammatory bowel disease
ICD: International Classification of Diseases
NPR: National Patient Register
SNOMED: Systematized Nomenclature of Medicine
UC: ulcerative colitis
