# Supplementary material for: Histologic Activity in Inflammatory Bowel Disease and Risk of Serious Infections: A Nationwide Study
Source: Clin Gastroenterol Hepatol. Author manuscript; Available in PMC 2025 Apr 1. (PMC10960698; doi:10.1016/j.cgh.2023.10.013)
Supplement: 1 [file NIHMS1941164-supplement-1.pdf]

Supplementary Materials

Supplementary Table 1 International Classification of Disease (ICD) codes and Systematized Nomenclature of Medicine (SNOMED) histology used in the definition of inflammatory bowel diseases (IBD).[1]

	**ICD-9**	**ICD-10**	**SNOMED codes [2]**
**Swedish National Patient Register**	**1987-1996**	**1997-**	**1965-**
Ulcerative colitis (UC)	556	K51	D6255<br>*or*<br>M41, M42, M43, M44, M463, or M47
Crohn's disease (CD)	555	K50	D6216<br>*or*<br>M41, M42, M43, M44, M463, or M47
IBD unclassified (IBD-U)	UC+CD [3]	UC + CD[3],<br>or K52.3	D6214<br>*or*<br>M41, M42, M43, M44, M463, or M47

[1] For details, please see: Olen O, Erichsen R, Sachs MC, Pedersen L, Halfvarson J, Askling J, et al. Colorectal cancer in Crohn's disease: a Scandinavian population-based cohort study. Lancet Gastroenterol Hepatol. 2020. doi:10.1016/S2468-1253(20)30005-4 and Olen O, Erichsen R, Sachs MC, Pedersen L, Halfvarson J, Askling J, et al. Colorectal cancer in ulcerative colitis: a Scandinavian population-based cohort study. Lancet. 2020;395:123-31. doi:10.1016/S0140-6736(19)32545-0

[2] Topography codes T65, T67-T68. For details on SNOMED classification, please see: Smedby B, Schiøler G. Health classifications in the Nordic countries. Historic development in a national and international perspective 2006. Edited by 76 NM-SCN. Nordisk Medicinalstatistisk Copenhagen: Komite; 2006.

[3] Participants with a mix of ICD codes for CD and UC were classified as IBD-U.

Supplementary Table 2 Definitions and diagnostic codes used to define ulcerative colitis and Crohn's disease according to the Montreal classification[1] since the start of the tenth revision of the International Classification of Diseases (ICD-10) (1997).

**Ulcerative colitis**	**Extent (E)**	**Diagnostic codes**
E1	Ulcerative proctitis	K51.2
E2	Left-sided	K51.3; K51.5
E3	Extensive (pancolitis)	K51.0
EX	Extent not defined	K51.4; K51.8; K51.9
**Crohn's disease**	**Location (L)**	**Diagnostic codes**
L1	Ileitis	K50.0
L2	Colonic	K50.1
L3/LX	Ileocolonic/location not defined	K50.8, K50.9

[1] Satsangi J, Silverberg MS, Vermeire S, et al. The Montreal classification of inflammatory bowel disease: controversies, consensus, and implications. Gut 2006;55:749-53

Supplementary Table 3 Surgery codes included in the definitions of inflammatory bowel disease (IBD)-related bowel surgery and perianal surgery (since 1964). Based on the Swedish translation of NOMESCO Classification of Surgical Procedures.[1]

Classification of surgical procedures	6th revision[1]	7th revision[1]
**Colectomy**		
**1) Subtotal colectomy with end ileostomy**		
Colectomy and ileostomy with closure of the rectum	4651	JFH10
Laparoscopic colectomy and ileostomy		JFH11
Other colectomy		JFH96
**2) Colectomy with IRA (ileorectal anastomosis)**		
Colectomy with ileorectal anastomosis	4650	JFH00
Laparoscopic colectomy with ileorectal anastomosis		JFH01
Ileorectal anastomosis		JFC40
Laparoscopic ileorectal anastomosis		JFC41
Closure of enterostomy with anastomosis to the rectum		JFG29
Closure of enterostomy with anastomosis to the colon		JFG26
**3) Partial colectomies**		
Right-sided colectomy	4641	JFB30, JFB31
Resection of the colon transversum	4643	JGB40, JFB41
Left-sided colectomy	4640	JFB43, JFB44
Resection of the sigmoid colon	4644	JFB46, JFB47
Other colon resection	4649	JFB50, JFB51
**4) Proctocolectomy with IPAA (ileal pouch-anal anastomosis)**		
Colectomy, rectal mucosectomy and ileoanal anastomosis *without* ileostomy.		JFH30
Colectomy, rectal mucosectomy and ileoanal anastomosis *and* ileostomy.		JFH33
Mucosectomy and ileoanal anastomosis after previous colectomy.	4654	JGB50
Extirpation of rectum or making of an ileoanal anastomosis after previous colectomy.		JGB60
**5) Continent ileostomy at time of colectomy**		
Proctocolectomy with continent ileostomy, “Kock”	4653	JFH40
Converting a conventional ileostomy to a continent ileostomy		JFG60
**6) Proctocolectomy**		
Proctocolectomy with ileostomy	4652	JFH20
**Other bowel surgery**	**1964-96**	**1997-**
Strictureplasty to the small bowel		JFA60
Strictureplasty to the colon		JFA63
Closure of small intestinal fistula		JFA76
Closure of colonic fistula		JFA86
Colonic and/or small bowel resection	4630, 4631, 4640-4649	JFB
Formation of stoma		JFF
Operations on intestinal stoma or reservoir		JFG
Other operation of the small bowel and/or colon	4660-4668, 4700-4739, 4790-4798	JFW96
Other laparoscopic operation of the small bowel and/or colon		JFW97

Rectal resection	4820-4828	JGB
**Perianal surgery**		
Perianal incision and drainage	4900	JHA00
Dilatation of the anal sphincter	4960	JHD00
Lay open or excision of perianal fistula	4920, 4922-4924	JHD20
Partial lay open or excision of perianal fistula (including seton placement)	4970-4971	JHD30
Completion lay open or excision of perianal fistula		JHD33
Excision of perianal fistula with advancement flap		JHD50
Occlusion of perianal fistula with collagen plug		JHD60
Occlusion of perianal fistula with fibrin glue		JHD63
Other anal or perianal surgery (e.g., examination under anesthesia)	4999	JHW96

[1] Socialstyrelsen (English: The National Board of Health and Welfare). Klassifikation av Vårdåtgärder [KVÅ-2015]. http://www.socialstyrelsen.se/klassificeringochkoder/atgardskoderkva. Accessed 17 Nov 2017.

Supplementary Table 4 Individuals diagnosed with inflammatory bowel disease (IBD) in 1990-2016 examined for the risk of serious infections during 0-<12 months with histological inflammation and 0-<12 months with histological remission.

Inclusion/exclusion	Histological inflammation		Histological remission		N patients	
	**N periods**	**N periods excluded**	**N periods**	**N periods excluded**	**Histological inflammation**	**Histological remission**
Individuals with IBD diagnosis in 1990-2016	85 034		41 767		51 294	28 774
**Exclusions within 5 years from index date[1]**						
Living outside of Sweden	83 476	1 558	41 087	680		
Any cancer recorded in the Cancer register[2]	80 258	3 218	39 411	1 676		
Tissue or organ transplantation[3]	79 776	482	39 143	268		
Immunodeficiency[3]	79 628	148	39 055	88		
HIV/AIDS[3]	79 590	38	39 039	16		
Hepatitis B/C[3]	79 313	277	38 885	154		
Tuberculosis[3]	79 242	71	38 859	26		
Any serious infection[4]	68 666	10 576	34 680	4 179		
**Total**	**68 666**	**16 368**	**34 680**	**7 087**	**43 523***	**24 479***

[1] Index date equals start of exposure as defined by date of biopsy showing histological inflammation or histological remission. Histological appearance was defined by SNOMED codes listed in Table S7. [2] Any cancer recorded in the Cancer Register, International Classification of Diseases (ICD)-7 code= 140-280; NOTE. In the Cancer Register, all ICD-8, -9, and -10 codes are back-translated to ICD-7. Details of the register can be found at: https://www.socialstyrelsen.se/en/statistics-and-data/registers/national-cancer-register/. [3] As defined using relevant ICD codes, listed in Table S5. [4] Defined as any inpatient infectious disease diagnosis, main or contributary, as listed in Table S6. * Total 55,626 unique individuals. AIDS, Acquired immunodeficiency syndrome; HIV, human immunodeficiency virus.

Supplementary Table 5. International Classification of Diseases (ICD) codes recorded in the National Patient Register for the exclusion of conditions with a susceptibility to serious infections.

**Exclusion criteria/Condition**	**ICD-8**	**ICD-9**	**ICD-10**
Tuberculosis	011-19	010-018, 320E	A15-A19, K23.0, K93.0
Hepatitis B/C [1]	-	070C-070G, 070X	B16, B170, B171, B180, B181, B182
HIV/AIDS [2]	-	079J, 279K (AIDS)	B20-24
Tissue or organ transplantation [3]	-	V42	Z94
Immunodeficiency	275	279	D80.0-D80.1, D80.3-D80.6, D80.8-D80.9, D81, D82 (*not* D823, D824A, D824B in SE), D83-D84, D89

[1] The Hepatitis C virus was first reported in 1989 and was therefore not included in ICD-8 or -9; Hepatitis B, discovered in 1965, was not specified in ICD-8. [2] Defined in the early 1980s, but first included in ICD-9. [3] Surgical/procedure codes: JJC, KAS, FQA, GDG, DR008, DR010, 6070, 5530, 5531, 5200, 5202, 3590 and 3085

Supplementary Table 6. International Classification of Diseases (ICD) codes recorded in the National Patient Register for the definition of serious infection overall, and by infectious disease category.

Serious infection category	ICD-7 code	ICD-8 code	ICD-9 code	ICD-10 code
**OVERALL**	Any of below	Any of below	Any of below	Any of below
**Sepsis**				
Sepsis	053	038	038	A40-41, R65.1
Septic shock			785F	R57.2
Anaerobic sepsis			038D	A41.4
Gram negative sepsis		038.80	038E	A41.5
Hemophilus influenzae sepsis				A41.3
Listeria sepsis				A32.7
Meningococcal sepsis	057.10	036.10,80, 97,99	036C-X	A39.2-9
Pneumococcal sepsis	053.20	038.20	038C	
Salmonella sepsis/(para)typhoid fever	040-041	001-002	002, 003B	A02.1
Staphylococcal sepsis (including TSS)	053.10	038.10	038B	A41.0-2, A48.3
Streptococcal sepsis	053.00	038.00	038A	A40
**Ear, nose and throat/respiratory**				
Bronchitis and bronchiolitis	500-501	466,99	466	J20, J21
Chronic obstructive lung disease with infection				J44.0
Inflammation and abscesses in salivary glands, mouth, tongue	537.40, 538.00	527.30, 528.30, 529.00	527C,D, 528D, 529A	K11.2-3, K12.2, K14.0
Influenza	480-483	470-474	487	J09-J11
Laryngitis, tracheitis and epiglottitis	474	464, 508.03	464	J04, J05
Mastoiditis, pertussis	392.00-01, 393.00,29	382.00,99 383.00,99	383A,C,X	H70.0, H70.2, H70.9, H75.0
Nasal abscess	517.01	508.01	478B	J34.0
Nasopharyngitis	470.99	460.99	460	J00
Other lower respiratory tract infection				J22

Otitis (incl external)	390, 391.00, 29, 392.00-01	380, 381.00,99, 382.00,99	380B, 382A,E,X	H60.0-3 H62.0-4, H66, H67.0-1
Parotitis	089	072	072	B26
Peritonsillar, pharyngeal and retropharyngeal abscess	511, 517.02-04	501.99, 508.02	475, 478C	J36, J39.0-1
Pertussis	056	033	033	A37
Pharyngitis	472	074.00-01, 462	074A, 462, 034A	B08.5, J02
Pleural empyema	518	510	510	J86
Pleuritis	519.10	511.10-20	511A,B, X	
Pneumonia (all: viral, bacterial, fungal)	096.83, 490, 491.10,19, 492-493	480.99 (virus) 481-484, 485.09, 486	480 (virus), 481, 482, 483, 484, 485, 48	J12 (Virus), J13, J14, J15, J16, J17, J18
Pulmonary abscess	521	513.99	006E, 513	A06.5, J85
Sinusitis including ethmoiditis	471	461	461	J01
Stomatitis (incl herpes simplex)	096.01, 096.60, 536	054.01, 079.40,	054C,078E	A69.0, A69.1B B00.2, B08.4,
Tonsillitis	051, 473	034.00, 463	034A, 463	J03
Unspecified respiratory tract infection				J98.7
Upper respiratory tract infection	475.99	465.99	465	J06
**Gastrointestinal/abdominal**				
Gastroenteritis – bacterial/protozoal	040-043, 045-048<br><br>Excl 046.10	001-004, 006-007 (excl 006.00), 008.00-008.30	001-004, 006A-C, W,X, 007, 008A-F	A00-04 A06-07 (excl A06.4-6)
Gastroenteritis - viral		008.80-98	008H-M	A08
Gastroenteritis – unspecified		009	008W, 009	A09
Hepatitis A			070A,B	B15
Hepatitis (unspecified/all virus)	092	070	070	B15-17, B19
Hepatitis, chronic (infectious)				B18
Intestinal abscess		569.00	569F	K63.0
Liver abscess (incl amoeba) /liver inf	582,046.10	006.00, 572.99	006D, 572A	A06.4, K75.0, K77.0
Perianal/anal abscess	575	566	566	K61
Peritonitis (abscesses)	576	567	567A,B,C,X	K65.0,9, K67
**Musculoskeletal/skin**				

Fasciitis				M72.6
Myositis	743.99	732.99, 074	728A	M60.0, M63.0-2
Osteomyelitis/osteitis	730.00, 29-30	720.00,29,30,39	730A,C,X	M86.0-2, M86.9, M90.0-2
Septic/infectious arthritis	720	710	711A	M00, M01
Spondylodiscitis				M46.2-3,5, M49.0-3
Synovitis				M65.0-1, M68.0
Cellulitis, lymphangitis and abscesses	690, 691.00-10, 691.99, 692, 693	680, 681.0-01,08-09, 682		L02, L03
Dermatitis, infectious	701.20	692.82		L30.3
Erysipelas	052	035.99	035	A46
Impetigo	695	684	684	L01
Lymphadenitis	694	683	683	L04
Other local infections of skin and subcutaneous tissue	698	686	006G, 686	A06.7, L08
Pilonidal cyst w. abscess			685A	L05.0
Staphylococcal scalded skin syndrome				L00
Varicella and herpes zoster	087-088	052, 053	052, 053	B01 B02
**Opportunistic**				
Pneumocystis		136.01	136D	B59
Cryptococcus	134.10			
Mycosis	131-134	110-117	110-118, 321A	B35-49, G02.1
Aspergillosis	134.50	117.3	117D,E	B44
Coccidiomycosis	133	114.99	114	B38
Candida	134.30	112	112	B37
Dermatophytosis and other superficial mycoses	131	110, 111	110,111	B35
**Other infections**				
Cystitis/urethritis	605.10,99, 607.00	595.00,09 597.00,09	595A,W,X, 597	N30.0, N30.8-9, N33, N34.0-1
Glomerular, tubulointerstitial disease (from infection) incl pyelonephritis	600.00-09, 600.28	590.10-14	078G, 590B,D,W,X	N08.0, N10, N12, N13.6, N16.0, A98.5

Hydrocele (infected)			603B	N43.1
Pelvic inf	626.00-10	567.00 616.00, 02		N74
Renal abscess	600.10	590.20	590C	N15.1
Urinary tract infection, unspecified	600.28	590.98-99, 599.02	599A	N15.9, N39.0, N29.1
Encephalitis	082, 087.20, 088.20, 089.20 096.04, 096.87, 093.20, 483.99	052.00, 054.04, 062-065, 072.01, 474.99<br>063	052B, 054D, 062-064, 323D,W,X<br>072C, 094X	A83-86, A89, B00.4, B01.1, B02.0, G04.2, G04.9, G05.0-2
Intracranial abscess	342	322	006F, 324A	A06.6 G06.0, G07
Intraspinal abscess	342.30	322.03	324B	G06.1-2
Meningitis - bacterial	057.00, 340,<br>087.2	320, 036.00	036A,B, 320A-D, H, W, X	A32.1, A39.0, G00-01
Meningitis - viral	096.52, 096.80, 87, 096.90-91, 340.39	045-046	047, 094B, 060-66, 054H, 053A, 049A, 048, 049, 053A, 054H, 321E, H	A87, B00.3, B02.1 G02.0
Protozoal diseases (incl malaria, toxoplasmosis)	110-117, 120-122	084-087, 130, 136.02,08	084, 085, 086, 130, 136C	(A06-7) B50-64, G02.8
Helminths	123-130	120-129	120-129	B65-B83
Zoonoses	044, 058-059, 062-063, 064.00-30, 082.00, 090, 091, 096.20	020-27, 039.00, 039.91-93, 060-064, 067-068, 089, 100, 079.30, 073.99	020-027, 060-066, 088, 100W,X, 078D, 073	A20-28, A32, A44, A70, A83-84, A92-99
Actinomycosis, nocardiosis	132.99	113.99	039	A42, A43
Bacterial infection (spec and unspec)	064.40, 064.99	039.90, 039.98	040, 041	A48 (excl A48.3), A49, B95-96
CMV		079.51-59	078F	B25
Diphtheria	055	032	032	A36
Fever		788.80		R50
Herpes simplex (extragenital)	096.01-09	054 excl 054.02	054 excl 054B, 321E	B00.0-1, B00.7-9, H19.1
HIV/AIDS			079J, 279K (AIDS)	B20-24
Infection of the eye	095, 096.03, 096.05, 096.19, 096.84. 370.00, 372, 376.00,	054.05, 076.99, 078, 362, 366.00-01, 368.00, 03,369.00-01	054E, 076, 077, 360A, 373B,C, 376A	A71, B00.5, B30, H00, H03, H05.0, H06.1, H10.0, H13.0-1, H19.0-2, H22.0, H32.0, H44.0

	378.00-01, 379.00-01			
Infection, unspecified	138.19	136.09	136W,X	B99
Leprosy	060	030	030	A30
Listeriosis		027.01-09	027A	A32
Mammary infection	621.00	611.00-01	611A, 680C	N61
Mononucleosis	093	075	075	B27
Mycobacterial inf (other)		031	031	A31
Peri-, myo- and endocarditis	430.00, 431.00-01, 432.00-01	074.20-21, 421.00	421A,B, 074C, 036E, 017W, 112W,002A	I30.1, I32.0-1, I33.0, I40.0, I41.0-2, I43.0, I52.0-1, B37.6, A39.5, A18.8, A01.0
Rheumatic fever (not chorea)	400-401	390-391	390-391	I00-01
Rickettsiosis	100-108	080-083	080-083	A75-79
Scarlatina	050	034.10-19	034B	A38
Spirochetal disease, incl borreliosis	070-074	088, 100, 101,102-103	087,101-104	A65-69
Splenic abscess		289.40		D73.3
Thymic abscess			254B	E06.0
Thyroid abscess			245A	E06.0
Tuberculosis	001-008, 010-019	011-19	010-018, 320E	A15-A19, K23.0, K93.0
Viral infection, spec + unspecified	096.51,59, 096.82, 096.86, 096.89, 096.99	074.10, 074.98-99, 079.80-99	074, 078W, 079A-X (excl 079J)	A88-89, B08.8, B09, B33, B34, B97

Supplementary Table 7 Systematized Nomenclature of Medicine (SNOMED) codes for histological inflammation and histological remission in inflammatory bowel disease

SNOMED code	Morphology	Definition of histological inflammation	Definition of histological remission
M00100<br>M00110	Normal<br>Normal	Absent	Present
M40000<br>M40400<br>M41000<br>M42100<br>M43000<br>M43030<br>M42000<br>M40460<br>M40700<br>M41700<br>M41740<br>M44000<br>M44700<br>M44900<br>M45020	Unspecified inflammation<br>Pustulous inflammation, unspecified<br>Acute inflammation<br>Acute and chronic inflammation<br>Chronic inflammation<br>Ulcer, chronic inflammation<br>Subacute inflammation<br>Inflammation with pus<br>Inflammation with necrosis<br>Inflammation with necrosis, abscess<br>Abscess<br>Granulomatous inflammation<br>Necrotizing granulomatous inflammation<br>Necrotizing fibrinous inflammation<br>Inflammation with granulation	Present[1]	Absent

[1] Minimum 1 histopathology (SNOMED) code for acute or chronic inflammation or ulceration/erosion.

Supplementary Table 8 International Classification of Diseases (ICD) codes for the definitions of chronic comorbidities.

**Diagnosis**	**ICD-9**<br>1987-1996	**ICD-10**<br>1997-
**Chronic comorbidities[1] for adjustment**		
Diabetes (type 1, type 2, and gestational)	250	E10-E14, O24
Hypertension	401-405	I10-I15
Asthma	493	J45
Autoimmune diseases[2]	242A, 242X, 244X, 245C, 245W, 579A, 696, 709A, 714, 710A	E035, E039, E050, E055, E059, E063, E065, K900, L40, L80, M05, M06, M123, M32

[1] Chronic comorbidities as recorded by index date which equals start of exposure as defined by date of biopsy showing histological inflammation or histological remission. [2] Autoimmune thyroid diseases, celiac disease, psoriasis, vitiligo, rheumatoid arthritis, and systemic lupus erythematosus.

Supplementary Table 9 Anatomical Therapeutic Chemical (ATC) codes of inflammatory bowel disease (IBD) treatment

Drug group	Substance	ATC-code
Immunomodulators	Azathioprine	L04AX0 1
	Mercaptopurine	L01BB02
	Methotrexate	L04AX03/L01BA01
Anti-tumor necrosis factor-alfa	Infliximab	L04AB02 (L04AA12 before 2008)
	Adalimumab	L04AB04 (L04AA17 before 2008)
	Golimumab	L04AB06
Other targeted therapies	Vedolizumab	L04AA33
	Ustekinumab	L04AC05
Corticosteroids, systemic	Betamethasone	H02AB01
	Dexamethasone	H02AB02
	Methylprednisolone	H02AB04
	Prednisolone	H02AB06
	Prednisone	H02AB07
	Hydrocortisone	H02AB09
	Cortisone	H02AB10
Corticosteroids, locally acting	Budesonide	A07EA06

Between January 1, 2006, throughout March 2016, there were no recorded use of Tofacitinib (ATC code L04AA29), Ozanimod (L04AA38) and Filgotinib (L04AA45).
